# Prehabilitation to Enhance Vascular Surgery Outcomes: A Narrative Review

**DOI:** 10.7759/cureus.70200

**Published:** 2024-09-25

**Authors:** Talal Altuwaijri

**Affiliations:** 1 Division of Vascular Surgery, Department of Surgery, College of Medicine, King Saud University, Riyadh, SAU

**Keywords:** teleprehabilitation, frailty, nutritional support, supervised exercise, multimodal prehabilitation, preoperative care, outcome, vascular surgery, prehabilitation

## Abstract

Prehabilitation, an emerging strategy, prepares patients for elective surgery by encouraging healthy behaviors, including physical exercise and healthy nutrition, while providing psychological support, to improve postoperative outcomes and foster healthier lifestyles. Despite growing interest, there is little research on prehabilitation. Specifically, studies involving prehabilitation in vascular surgery are heterogeneous with small sample sizes. This review aimed to investigate the reported positive impact of prehabilitation on vascular surgery patients, discuss prehabilitation models, highlight prehabilitation program-associated challenges, and suggest appropriate interventions. Prehabilitation improves physical fitness, reduces postoperative complications, and enhances overall recovery. Multimodal prehabilitation programs can positively impact vascular surgery patients, with benefits including improved cardiovascular fitness, reduced postoperative complications, shorter postoperative hospital stays, enhanced overall recovery, and improved quality of life. The currently reported prehabilitation programs are heterogeneous, with limitations regarding patient adherence and lack of long-term outcomes, posing challenges to their widespread adoption. Overall, prehabilitation shows promise for improving vascular surgery outcomes and fostering long-term healthy behaviors. The systematic implementation of prehabilitation in vascular surgery care pathways, overcoming reported limitations, and integrating multimodal prehabilitation into routine preoperative care hold potential benefits. This review underscores the need for high-quality research to establish best practices in prehabilitation and integrate them into the standard of care for vascular surgery patients.

## Introduction and background

Prehabilitation refers to the process of maintaining and enhancing an individual's physical functioning and capacity to withstand physiological stressors associated with an intervention [1]. Prehabilitation is a vital component of the healthcare journey that commences with referral of the patient for surgery and continues through their recovery, enhancing their resilience to surgery, promoting the success of their postoperative rehabilitation, encouraging the adoption of long-term healthy lifestyle behaviors, and leading to improved long-term health outcomes [2] in preparing for their procedure by encouraging the adoption of healthy behaviors, such as regular physical exercise, nutritional optimization, patient health education, providing psychological support, and controlling comorbidities. This is done to prepare patients for the physical and psychological stresses associated with the surgical procedure and to improve postoperative outcomes. The primary objective of prehabilitation is to enhance the physical, metabolic, and psychosocial reserves of surgical patients, thereby preparing them for their surgical intervention. It is also aimed at reversing frailty and improving functional capacity, which are crucial for more favorable surgical outcomes [1]. The beneficial effects of prehabilitation may vary depending on the type of surgical population, timing of initiation of the intervention, duration of the program, and patient adherence to the program [3]. Recent case studies and clinical applications have demonstrated the practical benefits and challenges of implementing prehabilitation programs in various surgical contexts [4].

This is particularly relevant in vascular surgery, where patients often possess multiple comorbidities, such as old age, hypertension, diabetes, cardiac disease, smoking, and a history of alcohol consumption, which complicate their surgical outcomes and make them more susceptible to postoperative surgical complications and prolonged recovery times, given the increase in the body's metabolic demands associated with vascular surgery procedures. Abdominal aortic aneurysms (AAAs) affect approximately 7.5% of males and 3% of females over 65 years of age [5], while 3%-5% of individuals aged 40 years or older are affected by peripheral arterial disease (PAD), which increases to 18% in those aged 70 years and above [6].

Frailty, defined as "a clinically recognizable state of increased vulnerability resulting from aging-associated decline in reserve and function across multiple physiologic systems such that the ability to cope with every day or acute stressors is comprised" [7], is common in vascular surgery patients. The use of tools and models to assess frailty during the preoperative phase can effectively identify the patient's surgical risk level, predict postoperative challenges [8,9], and improve their outcomes through patient-specific targeted interventions. Furthermore, the effective management of comorbidities, including glycemic control, blood pressure and cholesterol management, and smoking and alcohol cessation programs, has been shown to significantly reduce postoperative complications and improve overall surgical outcomes​​ [5,10-12].

The positive impact of prehabilitation in various surgical fields has been documented in recent years, and vascular surgery patients could potentially benefit considerably from prehabilitation programs. This narrative review aims to provide a comprehensive understanding of the concept of prehabilitation for surgical patients and an updated overview of recent studies related to prehabilitation in vascular surgery.

## Review

Prehabilitation in vascular surgery

Vascular surgery patients are often high-risk and frail, making them suitable candidates for prehabilitation to potentially reduce postoperative complications. It is essential to address frailty and physical inactivity because they are linked to suboptimal postoperative outcomes. Clinicians should aim to reverse frailty to enhance patient outcomes [8-11]. Prehabilitation programs are based on the premise that patients with greater functional capability will be better able to tolerate surgical intervention stressors and recover more rapidly [13,14]. Earlier interventions, ideally at the time of referral, are considered to be better for improving patient outcomes. Therefore, the prehabilitation process should effectively begin at the primary care stage, concurrent with the referral of the patient to the vascular surgery service. Early interventions can potentially improve the patient's overall health condition and healthcare outcomes, even when their suitability for the surgical intervention is uncertain [2,11]. The duration of prehabilitation programs reported in the literature varies, with some studies reporting a four-week duration and others reporting longer periods [15,16]. Tew et al. reviewed the impact of preoperative exercise training in adults undergoing elective major vascular surgery. The study revealed that preoperative exercise training reduced the likelihood of postoperative cardiac and renal complications, showing significant improvements in postoperative outcomes, including reduced postoperative complications and hospital length of stay [17].

Prehabilitation improves physical functioning and quality of life measures, in addition to improving aerobic capacity, maximum walking distance, and pain-free walking distance in vascular surgery patients [18]. Patients who participated in prehabilitation programs reported significant improvement in health-related quality of life within the context of Enhanced Recovery After Surgery (ERAS) protocols, including enhancing their overall health, physical capacity, and mental health condition [1,19,20]. Santa Mina et al. conducted a systematic review and meta-analysis on the concept of total-body prehabilitation, indicating its effectiveness in improving postoperative complications, length of hospital stay, and pain following surgery resulting in significant cost savings [21]. While there is strong evidence supporting the effectiveness of prehabilitation in cardiac surgery, more research is required to further explore the evidence around it in oncology surgery [10,22-25]. High-quality evidence remains necessary to establish the prehabilitation impact on vascular surgery patients.

To motivate inactive patients to engage in regular social and physical healthy activities, vascular surgery services must expand their patient management domains and implement strategies beyond traditional vascular surgery practice [26].

Components of prehabilitation programs

Prehabilitation programs typically comprise the following components: exercise training, nutritional support, and psychological counseling and support. Multimodal prehabilitation is a model where these components are combined.

Exercise Training

Exercise prehabilitation typically includes aerobic exercises (e.g., cycling and walking) and resistance training to improve cardiovascular fitness, muscle strength, and overall physical condition and promote recovery after surgery [19,20,27,28]. The current research demonstrates that physical fitness positively impacts most aspects of health and healthcare [29]. Engaging in physical activity prior to surgery, including aerobic and resistance training, can improve overall fitness and better prepare patients for the demands of the surgical procedure. Studies have demonstrated that preoperative exercise can correlate with better postoperative outcomes, reducing postoperative complications and hospital stays after major elective surgical procedures [23,30-32]. There is growing evidence supporting the benefits of preoperative exercise in reducing complications and improving recovery outcomes for surgical patients [5]. Research suggests that supervised exercise is more effective than unsupervised exercise in increasing overall functional capacity and pain-free walking distance [18]. A mixed approach involving one supervised exercise session per week can aid patients in achieving the benefits of supervised exercise while maintaining effective compliance and controlling costs [18]. Barakat et al. conducted a randomized controlled trial in which 124 patients scheduled for open and endovascular AAA repair were randomized to either a six-week preoperative supervised exercise program (SEP) or standard treatment. Patients who participated in the exercise program before surgery had a reduction in their hospital stay by one day and a significantly reduced postoperative complications rate compared to those in the standard treatment group. Patients who adhered to more than 75% of the prescribed exercise sessions in this program experienced a significantly lower incidence of postoperative complications [27]. However, there is considerable heterogeneity in the existing literature regarding the nature of prehabilitation exercise protocols, and improved design of training variables could enhance overall outcomes [33].

Total body prehabilitation and high-intensity training programs are areas of potential interest that open up prospects for future research in prehabilitation programs for vascular surgery patients [10,21,34]. Bauer et al. conducted a pilot study involving a small number of patients and reported that a four-week prehabilitation exercise program significantly increased the number of endothelial progenitor cells in circulation. This suggests that exercise prehabilitation not only improves physical capacity but also induces beneficial cellular changes that enhance the body's regenerative capacity, potentially leading to reduced surgical complications and improved recovery [15].

Nutritional Support

Of individuals with PAD, 26% suffer from malnutrition, which has been correlated with poorer postoperative outcomes, exacerbated systemic inflammatory responses, prolonged postoperative hospital stays, muscular weakness, impaired immunological function, wound healing problems, and extended intensive rehabilitation following major vascular surgery [8,18]. Nutritional supplementation objectives include providing the additional energy required for exercise program activities, enhancing tissue repair, and supporting the healing process following the catabolic impact of the surgical intervention [6]. Nutritional support, particularly sufficient protein intake, ensures patients are nutritionally optimized for surgery. Proper nutrition is crucial for promoting healing and has been shown to independently lower postoperative complications and shorten hospital length of stay [10,31,35]. Effective glycemic control is crucial for reducing the risks of postoperative infections, limb loss, and mortality in patients undergoing vascular surgery [8]. A program of 5-7 days of enteral nutritional supplementation provided a 50% reduction in major postoperative complications [29]. Most patients can be effectively supported through appropriate professional dietary guidance, along with the inclusion of a nutritional whole protein liquid standard supplement [36]. Coca-Martinez et al. reported a 69% patient adherence to nutritional sessions [18].

Psychological Counseling and Support

In addition to their physiological readiness, patients require mental and emotional preparation to cope with the demands of the surgical intervention [37]. Psychological interventions, such as relaxation techniques, mental resilience training, cognitive behavioral therapy, and stress management aid in managing anxiety and stress related to surgery. Mental health support and coping strategies are essential for reducing preoperative anxiety and improving postoperative recovery and postoperative psychological outcomes [6,10,20,38].

Multimodal Prehabilitation

Multimodal prehabilitation is a combined multidisciplinary intervention designed to optimize the patient's readiness in preparation for a surgical procedure or a major stressor. Programs include structured physical exercise programs, nutritional support, psychological counseling, medical optimization, and smoking and alcohol cessation programs [18,20]. Multimodal prehabilitation effectively improved perioperative functional capacity, functional recovery, and quality of life and significantly reduced postoperative complications, rehabilitation needs, and hospital length of stay, particularly in frail patients undergoing major surgeries [8,19,31]. The combination of physical, nutritional, and psychological interventions in a structured prehabilitation program demonstrated significant effectiveness in enhancing surgical outcomes compared to unimodal approaches. Multimodal prehabilitation is a promising and practical approach to enhance the functional capacity and quality of life of patients with PAD who experience severe life-limiting intermittent claudication or those with advanced challenging infrainguinal disease or failed revascularization surgical attempts [32]. Furthermore, Coca-Martinez et al. conducted an in-trial pilot randomized controlled trial and demonstrated that multimodal prehabilitation enhanced functional outcomes, walking capacity, and disease-specific quality of life in patients with PAD. The effects of this impact were sustained for up to one year after the intervention [18].

Prehabilitation and cardiovascular fitness

Cardiovascular fitness is a crucial factor in determining surgical outcomes particularly in higher-risk patients or procedures. Poor cardiovascular health is associated with increased surgical risks and complications. Prehabilitation aims to improve cardiovascular fitness through structured exercise programs including aerobic and resistance training. Preoperative physical fitness assessment can be challenging to measure, but cardiopulmonary exercise testing (CPET) is a valuable tool for evaluating a patient's fitness level and measuring exercise capacity. CPET can predict postoperative outcomes and surgical risks, providing prognostic information for postoperative outcomes, particularly in vascular surgery patients. CPET assesses the physiological response of the entire body to structured physical activity and provides an objective evaluation of the patient's physical capacity, including functional capacity and physical fitness. CPET provides a global evaluation of the integrated systemic metabolic response of the pulmonary and cardiovascular systems and permits the examination of the causes of physical challenges when exercise capacity is diminished [39]. CPET has been found to independently predict the 30-day outcome and mortality in addition to estimating three-year survival rates [8]. Reduced physical aerobic fitness, demonstrated by an anaerobic threshold (AT) below 10.2 mL/kg/minute, peak oxygen consumption (VO2 peak) below 15 mL/kg/minute, and low ventilatory efficiency consistent with a minute ventilation/carbon dioxide production (VE/VCO2) of less than 42, has been shown to independently predict reduced survival rates following open and endovascular aortic surgery [10]. Improved preoperative functional reserve in terms of CPET parameters (e.g., peak oxygen uptake (VO2 peak) and oxygen uptake anaerobic threshold (VO2 AT)) and the six-minute walk test (6MWT) results have been linked to better surgical outcomes as regards the length of postoperative hospital stay, pulmonary and cardiac complications, and mortality after major vascular surgeries; this makes the preoperative phase a valuable window to enhance overall surgical outcomes and reduce postoperative complications. In some settings, CPET is becoming a part of the routine planning process for the decision-making on the patient's fitness for the surgical intervention [3,12,18,19,23,30,35].

Studies have demonstrated that even a modest improvement in cardiovascular fitness can lead to significant benefits. For example, each 1 mL/kg/minute increase in VO2 peak has been linked to a 15% reduction in the overall and cardiovascular risk of mortality in patients with coronary artery disease [40]. Myers et al. found that exercise capacity provided a significant prediction of mortality in patients referred for exercise testing and suggested that exercise capacity significantly predicted mortality more effectively, thereby emphasizing the importance of cardiovascular fitness [41].

Structured trials have demonstrated that supervised exercise testing can help enhance and possibly guide the surgical decision effectively regarding the type of surgical procedure (endovascular or open surgical repair) or the postoperative level of healthcare requirements in patients scheduled for AAA surgical interventions [8].

Clinical evidence of the impact of prehabilitation in vascular surgery

Most evidence on prehabilitation in vascular surgery comes from small, heterogeneous studies, which makes it challenging to reach definitive conclusions. High-quality research specifically focused on prehabilitation for vascular surgery patients is required [10]. Several studies and systematic reviews have evaluated the impact of prehabilitation on vascular surgery outcomes for different vascular interventions.

Abdominal Aortic Aneurysm (AAA) Repair

The preoperative functional condition of the patient is crucial in predicting their capacity to tolerate the stresses associated with AAA surgical repair [10]. Barakat et al. conducted a randomized controlled trial in which patients undergoing AAA repair were assigned to either a standard care group or a six-week supervised exercise program (SEP) group. The SEP group showed reduced postoperative cardiovascular, pulmonary, and renal complications, shorter hospital stay, and improved fitness level compared to the control group [27]. Similarly, Sethi et al. conducted a randomized controlled trial on patients planned for open or endovascular AAA repair to either a six-week preoperative SEP or standard management. The results revealed that SEP preceding elective AAA repair significantly improved overall cardiac, pulmonary, and renal complications. Additionally, SEP improved fitness and long-term mortality after five years of follow-up [30]. The UK-based Endovascular Aneurysm Repair (EVAR trial 2) study recommended that vascular surgery teams should focus on improving patient fitness levels before surgery [42]. However, other reviews suggested that there is very low certainty evidence on the benefits of prehabilitation exercise therapy for reducing 30-day mortality, as well as pulmonary, cardiac, and renal complications. This was primarily due to the low certainty evidence and the small number of trials available to date [43]. The inclusion of prehabilitation programs as a standard component of the surgical healthcare pathway for patients undergoing AAA surgery remains a topic of ongoing debate. However, it is widely acknowledged that the period between diagnosis and the surgical intervention presents a valuable opportunity to proactively manage risk factors and enhance the patient's overall health and physical capacity [10].

Peripheral Arterial Disease

Patients with lower limb PAD face unique challenges that impact their ability to achieve presurgical exercise program goals. Intermittent claudication results in a significant limitation in walking capacity and general physical activity, considerably affecting their quality of life [10]. Recently, Coca-Martinez et al. conducted a pilot randomized controlled trial comparing the impact of multimodal prehabilitation including supervised exercise with unsupervised walking advice for patients with PAD who are candidates for endovascular revascularization. The study concluded that multimodal prehabilitation improved the quality of life and functional walking capacity of the patients compared with unsupervised unstructured walking advice. These outcomes were maintained over the one-year follow-up period, suggesting that supervised exercise should be considered a fundamental component of any prehabilitation program for patients with intermittent claudication, as it greatly enhances the patient's adherence to exercise and results in more optimal outcomes than unsupervised exercise [18]. However, prior reviews found no randomized controlled trials available to definitively confirm the effectiveness of prehabilitation in improving postoperative outcomes in patients with PAD [6]. A case series involving a small number of patients assessed the effect of a 12-week multimodal prehabilitation program including supervised and home-based exercises on the quality of life and functional capacity in patients with PAD. The program achieved promising improvements in physical functional capacity and overall quality of life, particularly in walking distance and pain onset [32].

Other Vascular Conditions

No trials involving prehabilitation interventions in patients diagnosed with carotid artery disease were found due to the nature of carotid artery disease presentation and management, as revascularization for carotid artery disease is not an ideal context to assess the potential impact of prehabilitation interventions [17].

Aragoncillo Sauco et al. demonstrated significant increases in venous caliber, arterial caliber, and arterial peak systolic velocity after an eight-week isometric exercise protocol in patients with end-stage renal disease requiring a new arteriovenous fistula for hemodialysis. The exercise group had a higher probability of distal arteriovenous fistula creation [44].

Limitations of prehabilitation programs and current evidence

Although prehabilitation programs show promise in improving patients' overall health condition and operative course, there are several challenges and limitations.

Heterogeneity of Prehabilitation Programs

Prehabilitation programs vary widely in their definitions, components, duration, intensity, delivery protocols, baseline assessment protocols, and outcome definitions, which makes it challenging to isolate the specific impact and cost-effectiveness of individual components. This makes it difficult to standardize protocols and compare outcomes across studies and justify funding and overall adoption of prehabilitation programs ​​to preoperative healthcare settings [3,26,45]. The heterogeneity of prehabilitation interventions and the varying methodologies among existing studies pose challenges to recommending prehabilitation programs as a standard of care to patients scheduled for major vascular surgery [10].

Importance of High Patient Adherence to Prehabilitation Programs

Ensuring consistent patient participation in prehabilitation processes can be challenging as it is influenced by numerous factors such as physical, behavioral, psychological, physiological, environmental, and social considerations [46]. Barriers challenging patients' participation in prehabilitation programs reported in the literature are related to pain hindering participation, waiting times, transportation difficulties, financial constraints, finding the time for exercise, program availability, and lack of interest and motivation. These factors can limit adherence to prehabilitation programs, highlighting the importance of high compliance and patient adherence rates for successful and effective prehabilitation. Reports indicate that less than 50% of patients demonstrate adherence due to lack of motivation and interest. However, patient adherence to prehabilitation programs can reach up to 84% with proper supervision, guidance, and support [18,38,47-49]. Programs need to be customized to individual patient needs and to local resources, which adds efficiency and sustainability to their design and implementation. It is fundamental to take into account the frequency, intensity, duration, type, volume, and advancement plan when planning the exercise intervention; this needs to be customized for each patient depending on their health condition and circumstances [10]. Research indicates that positive behavioral modification can be achieved by collaborating with patients to set exercise goals, define exercise challenges, and maintain exercise diaries [50]. Fostering internal motivation and encouraging independence and confidence in addition to using personally developed exercise diaries as well as actively engaging patients and encouraging them to play active roles within the exercise program are crucial to enhancing patient adherence to prehabilitation exercise programs [51].

Evidence of Long-Term Outcomes Benefits of Prehabilitation Programs

The long-term impact of prehabilitation on morbidity and mortality in vascular surgery intervention patients has not been thoroughly examined in the current literature [1], with many studies focusing on short-term outcomes. Long-term data to understand the sustained benefits of prehabilitation are required. Long-term follow-up studies are essential to determine the impact of prehabilitation on overall outcome, survival, and quality of life.

Commitment and Funding to Sustainable Prehabilitation Programs

Prehabilitation programs are resource intensive, and ensuring key stakeholder commitment and sufficient funding can be a significant challenge. Regular monitoring and outcome assessment are required to justify the continuity of support for prehabilitation programs and their sustainability [2].

Future directions and recommendations

The New South Wales (NSW) Agency for Clinical Innovation in NSW, Australia, issued a publication prepared by the Surgical Services Taskforce in September 2022 on prehabilitation key principles for preparing patients for surgery, identifying the five key principles for effective prehabilitation: meeting the local community requirements, including interventions that have satisfactory evidence in literature; establishing referral pathways and assessment protocols; ensuring patient engagement and preoperative education to ensure the partnership of patients in their care; and supporting prehabilitation program sustainability. The publication highlighted the enablers for effective implementation as proper governance and clinical oversight, appropriate resourcing, monitoring, and evaluation including commitment to gathering and evaluating relevant data, which are vital for evaluating the program's performance and impact [2].

Committed leadership and multidisciplinary working teams, engaged patients and stakeholders, and sustainable funding resources are all fundamental elements of an effective, stable prehabilitation program [10]. To optimize the benefits of prehabilitation in vascular surgery, several actions can be taken.

Standardization of Prehabilitation Programs

Developing standardized protocols for prehabilitation can aid in ensuring consistency and improve outcome assessment while establishing evidence-based best practices [21,35]. Standardized protocols should include guidelines on exercise type, intensity, frequency, and duration, as well as nutritional and psychological support. Standardizing assessment tools and protocols across services is crucial to ensure consistency [2]. Prehabilitation protocols must consider individualized patient needs based on baseline assessments, including risk stratification assessment to determine high-risk individuals who would benefit most from the prehabilitation interventions [10,45]. Pending the development of evidence-based, structured, and standardized prehabilitation protocols, healthcare teams can create individualized, patient-focused prehabilitation programs that appropriately address all prehabilitation dimensions [52].

Integration of Prehabilitation Programs Into Routine Vascular Surgery Standard of Care

Prehabilitation should be integrated into routine preoperative care to maximize its benefits. Clear referral pathways and assessment mechanisms are essential for the effective implementation of prehabilitation programs, ensuring patients are appropriately screened and referred. This involves integrating prehabilitation into the patient's surgical care pathways and standard healthcare models, which requires coordination and collaboration between surgeons, physiotherapists, dietitians, and psychologists. Multidisciplinary teams can provide comprehensive prehabilitation programs tailored to individual patient needs at the appropriate time [2,10]. The success of social prescribing in primary care, such as guidance on participating in community running and walking groups, shows promise in organizing physical activity in a more inclusive manner [26].

Addressing Barriers to Patient Adherence

It is essential to implement strategies to improve patient adherence, such as providing transportation support, remote supervision options such as teleprehabilitation​​, and financial assistance. Ensuring that prehabilitation programs are accessible and affordable is crucial for their success. Effective strategies to improve adherence include patient education, motivational interviewing, logistical support, and the understanding of exercise program advantages, which can help support the commitment and adherence of patients to their prehabilitation programs [48], in addition to increasing the awareness and commitment among healthcare leaders, providers, and multidisciplinary coordination [10].

Home-based prehabilitation: Home-based exercise can help overcome barriers faced by patients in prehabilitation programs and is endorsed by clinical practice guidelines as an effective treatment for patients with PAD [47]. Waite et al. reported that a home-based prehabilitation program for frail patients awaiting coronary artery bypass graft and valve surgery improved functional ability and reduced hospital length of stay [12]. Patients generally prefer an exercise program that is home-based, with one supervised session per week [48]. A study by Coca-Martinez et al. showed that patient adherence to home-based exercise programs was 90% [18]. Home-based programs may be more appropriate for frail patients or those with logistical challenges, emphasizing the potential benefits of reducing transport time and increasing patient comfort. A combined home and on-site supervised program can be enhanced using remote supervision options such as teleprehabilitation​ to maximize the benefits of the prehabilitation program.

Patient engagement and education: The process of engaging patients as active participants in their own healthcare through preoperative education, shared decision-making, and prehabilitation intervention planning involves providing information regarding their surgery, recovery process, and the importance of maintaining an active lifestyle through a healthy diet, physical activity, and smoking and alcohol cessation [2,38]. Teleprehabilitation has enabled patients who are unable or unwilling to engage in the prehabilitation process to participate remotely, thereby reducing their financial burden, and has the potential to be cost-effective for the healthcare system, resulting in wider patient participation and engagement [10,49]. Wu et al. recommend setting patient-specific plans and suitable and attainable prehabilitation goals, as well as teaching patients the necessary skills to self-manage their health when designing a prehabilitation program [49].

Research

Current research focuses on optimizing prehabilitation protocols, understanding patient adherence factors, and identifying the most effective components of prehabilitation programs. However, to date, significant limitations exist regarding the conclusions and meta-analyses of prehabilitation data, such as heterogeneity with high variability in perioperative protocols, prehabilitation models, and outcome assessment standards. Studies generally involve small sample cohorts and lack high-quality methodological design, with significant risk of bias [10]. Further research and large-scale, high-quality studies are required to identify the most effective components of prehabilitation programs, assess their cost-effectiveness, and evaluate their long-term impact on surgical outcomes. Future studies should focus on determining the optimal form, timing, intensity, and duration of prehabilitation programs, as well as exploring the feasibility of incorporating the programs into routine clinical practice [10,12,43,45]. The improved strength of evidence for prehabilitation demands high-quality clinical trials with strong methodological design and reporting to address crucial gaps in current knowledge limitations and utilize standardized common definitions and outcome sets [3,5].

## Conclusions

Prehabilitation is promising and has the potential to significantly enhance outcomes for vascular surgery patients by improving their physical and mental health before surgery. Patient fitness positively impacts almost every aspect of healthcare. Research continues to confirm the relationship between fitness level and enhanced perioperative outcomes. Multimodal prehabilitation programs have been shown to reduce postoperative complications, shorten hospital stays, improve overall recovery, and promote sustainable healthy behavioral changes. Current evidence supports the effectiveness of prehabilitation in cardiac and oncology surgery, yet there is a need for high-quality evidence of its impact in vascular surgery.

The integration of standardized prehabilitation protocols into routine preoperative care could make this approach a key component of vascular surgery patient preparation. Ongoing high-quality research and efforts to address the optimal program design, implementation challenges, and program barriers will be essential to realize the full benefits of prehabilitation and effectively incorporate sustainable protocols into vascular surgery patient care.

## References

[1] Drudi LM, Tat J, Ades M (2019). Preoperative exercise rehabilitation in cardiac and vascular interventions. J Surg Res.

[2] (2024). Prehabilitation: key principles for preparing patients for surgery. https://aci.health.nsw.gov.au/__data/assets/pdf_file/0005/743360/ACI-Prehabilitation-key-principles-for-preparing-patients-for-surgery.pdf.

[3] McIsaac DI, Gill M, Boland L (2022). Prehabilitation in adult patients undergoing surgery: an umbrella review of systematic reviews. Br J Anaesth.

[4] Cui HW, Turney BW, Griffiths J (2017). The preoperative assessment and optimization of patients undergoing major urological surgery. Curr Urol Rep.

[5] Wee IJ, Choong AM (2020). A systematic review of the impact of preoperative exercise for patients with abdominal aortic aneurysm. J Vasc Surg.

[6] Palmer J, Pymer S, Smith GE, Harwood AE, Ingle L, Huang C, Chetter IC (2020). Presurgery exercise-based conditioning interventions (prehabilitation) in adults undergoing lower limb surgery for peripheral arterial disease. Cochrane Database Syst Rev.

[7] Xue QL (2011). The frailty syndrome: definition and natural history. Clin Geriatr Med.

[8] Czobor NR, Lehot JJ, Holndonner-Kirst E, Tully PJ, Gal J, Szekely A (2019). Frailty in patients undergoing vascular surgery: a narrative review of current evidence. Ther Clin Risk Manag.

[9] Amrock LG, Deiner S (2014). The implication of frailty on preoperative risk assessment. Curr Opin Anaesthesiol.

[10] Shovel L, Morkane C (2022). Prehabilitation for vascular surgery patients: challenges and opportunities. Can J Cardiol.

[11] Benson R, McGregor G, Shehata M, Imray C (2019). Optimising fitness for major vascular surgery. BMJ.

[12] Waite I, Deshpande R, Baghai M, Massey T, Wendler O, Greenwood S (2017). Home-based preoperative rehabilitation (prehab) to improve physical function and reduce hospital length of stay for frail patients undergoing coronary artery bypass graft and valve surgery. J Cardiothorac Surg.

[13] Brown R, Bozeman P (2023). Prehabilitation program for patients with chronic limb threatening ischemia: lessons learned from a systematic review and metanalysis evaluating prehabilitation programs in general abdominal surgery. J Vasc Nurs.

[14] Myers J, Niebauer J, Humphrey R (2021). Prehabilitation coming of age: implications for cardiac and pulmonary rehabilitation. J Cardiopulm Rehabil Prev.

[15] Bauer CJ, Findlay M, Koliamitra C (2022). Preoperative exercise induces endothelial progenitor cell mobilisation in patients undergoing major surgery - a prospective randomised controlled clinical proof-of-concept trial. Heliyon.

[16] Tew GA, Batterham AM, Colling K (2017). Randomized feasibility trial of high-intensity interval training before elective abdominal aortic aneurysm repair. Br J Surg.

[17] Tew GA, Caisley K, Danjoux G (2022). Preoperative exercise training for adults undergoing elective major vascular surgery: a systematic review. PLoS One.

[18] Coca-Martinez M, Girsowicz E, Doonan RJ (2024). Multimodal prehabilitation for peripheral arterial disease patients with intermittent claudication-a pilot randomized controlled trial. Ann Vasc Surg.

[19] Jain SR, Kandarpa VL, Yaow CY (2023). The role and effect of multimodal prehabilitation before major abdominal surgery: a systemic review and meta-analysis. World J Surg.

[20] Mesnard T, Dubosq M, Pruvot L, Azzaoui R, Patterson BO, Sobocinski J (2023). Benefits of prehabilitation before complex aortic surgery. J Clin Med.

[21] Santa Mina D, Clarke H, Ritvo P (2014). Effect of total-body prehabilitation on postoperative outcomes: a systematic review and meta-analysis. Physiotherapy.

[22] Rouleau CR, Chirico D, Hauer T, Kidd W, Arena R, Aggarwal SG (2022). An observational study examining utilization of prehabilitation and its association with postoperative cardiac rehabilitation participation and risk factors following coronary artery bypass grafting. Int J Cardiol.

[23] Steinmetz C, Bjarnason-Wehrens B, Baumgarten H, Walther T, Mengden T, Walther C (2020). Prehabilitation in patients awaiting elective coronary artery bypass graft surgery - effects on functional capacity and quality of life: a randomized controlled trial. Clin Rehabil.

[24] Steinmetz C, Bjarnason-Wehrens B, Walther T, Schaffland TF, Walther C (2023). Efficacy of prehabilitation before cardiac surgery: a systematic review and meta-analysis. Am J Phys Med Rehabil.

[25] Sliwinski S, Werneburg E, Faqar-Uz-Zaman SF (2023). A toolbox for a structured risk-based prehabilitation program in major surgical oncology. Front Surg.

[26] Mastracci TM (2022). Prehabilitation before surgery: a (social) prescription for change. Eur J Vasc Endovasc Surg.

[27] Barakat HM, Shahin Y, Khan JA, McCollum PT, Chetter IC (2016). Preoperative supervised exercise improves outcomes after elective abdominal aortic aneurysm repair: a randomized controlled trial. Ann Surg.

[28] Zheng YT, Zhang JX (2020). Preoperative exercise and recovery after cardiac surgery: a meta-analysis. BMC Cardiovasc Disord.

[29] Levett DZ, Edwards M, Grocott M, Mythen M (2016). Preparing the patient for surgery to improve outcomes. Best Pract Res Clin Anaesthesiol.

[30] Sethi S, Ravindhran B, Long J (2024). A preoperative supervised exercise program potentially improves long-term survival after elective abdominal aortic aneurysm repair. J Vasc Surg.

[31] Gillis C, Buhler K, Bresee L (2018). Effects of nutritional prehabilitation, with and without exercise, on outcomes of patients who undergo colorectal surgery: a systematic review and meta-analysis. Gastroenterology.

[32] Coca-Martinez M, Carli F, Gill HL (2021). Multimodal prehabilitation to improve quality of life and functional capacity in peripheral arterial disease: a case series. Arch Rehabil Res Clin Transl.

[33] Orange ST, Northgraves MJ, Marshall P, Madden LA, Vince RV (2018). Exercise prehabilitation in elective intra-cavity surgery: a role within the ERAS pathway? A narrative review. Int J Surg.

[34] Tew GA, Ayyash R, Durrand J, Danjoux GR (2018). Clinical guideline and recommendations on pre-operative exercise training in patients awaiting major non-cardiac surgery. Anaesthesia.

[35] Gillis C, Li C, Lee L (2014). Prehabilitation versus rehabilitation: a randomized control trial in patients undergoing colorectal resection for cancer. Anesthesiology.

[36] Melnyk M, Casey RG, Black P, Koupparis AJ (2011). Enhanced recovery after surgery (ERAS) protocols: time to change practice?. Can Urol Assoc J.

[37] Levett DZ, Grimmett C (2019). Psychological factors, prehabilitation and surgical outcomes: evidence and future directions. Anaesthesia.

[38] Olsen DB, Pedersen PU, Noergaard MW (2021). Prehabilitation before elective coronary artery bypass grafting surgery: a scoping review protocol. JBI Evid Synth.

[39] Levett DZ, Jack S, Swart M (2018). Perioperative cardiopulmonary exercise testing (CPET): consensus clinical guidelines on indications, organization, conduct, and physiological interpretation. Br J Anaesth.

[40] Keteyian SJ, Brawner CA, Savage PD (2008). Peak aerobic capacity predicts prognosis in patients with coronary heart disease. Am Heart J.

[41] Myers J, Prakash M, Froelicher V, Do D, Partington S, Atwood JE (2002). Exercise capacity and mortality among men referred for exercise testing. N Engl J Med.

[42] (2005). Endovascular aneurysm repair and outcome in patients unfit for open repair of abdominal aortic aneurysm (EVAR trial 2): randomised controlled trial. Lancet.

[43] Fenton C, Tan AR, Abaraogu UO, McCaslin JE (2021). Prehabilitation exercise therapy before elective abdominal aortic aneurysm repair. Cochrane Database Syst Rev.

[44] Aragoncillo Sauco I, Hevia C, Manzano Grossi S (2021). Effect of preoperative exercise on vascular caliber and maturation of arteriovenous fistula: the physicalfav trial, a randomized controlled study. J Nephrol.

[45] Cui H, Fairer K (2021). An introduction to surgical prehabilitation. J Nuffield Dep Surg Sci.

[46] Wynter-Blyth V, Moorthy K (2017). Prehabilitation: preparing patients for surgery. BMJ.

[47] Cetlin MD, Polonsky T, Ho K (2023). Barriers to participation in supervised exercise therapy reported by people with peripheral artery disease. J Vasc Surg.

[48] Ferreira V, Agnihotram RV, Bergdahl A, van Rooijen SJ, Awasthi R, Carli F, Scheede-Bergdahl C (2018). Maximizing patient adherence to prehabilitation: what do the patients say?. Support Care Cancer.

[49] Wu F, Laza-Cagigas R, Rampal T (2022). Understanding patients’ experiences and perspectives of tele-prehabilitation: a qualitative study to inform service design and delivery. Clin Pract.

[50] French DP, Olander EK, Chisholm A, Mc Sharry J (2014). Which behaviour change techniques are most effective at increasing older adults' self-efficacy and physical activity behaviour? A systematic review. Ann Behav Med.

[51] Collado-Mateo D, Lavín-Pérez AM, Peñacoba C (2021). Key factors associated with adherence to physical exercise in patients with chronic diseases and older adults: an umbrella review. Int J Environ Res Public Health.

[52] Bargnes V 3rd, Davidson S, Talbot L, Jin Z, Poppers J, Bergese SD (2024). Start strong, finish strong: a review of prehabilitation in cardiac surgery. Life (Basel).

